# Contributions and challenges of healthcare financing towards universal health coverage in Ethiopia: a narrative evidence synthesis

**DOI:** 10.1186/s12913-022-08151-7

**Published:** 2022-07-05

**Authors:** Ayal Debie, Resham B. Khatri, Yibeltal Assefa

**Affiliations:** 1 Department of Health Systems and Policy, Institute of Public Health, College of Medicine and Health Sciences, University of Gondar, University of Gondar, P.O. Box: 196, Gondar, Ethiopia; 2 School of Public Health, the University of Queensland, Brisbane, Australia

**Keywords:** Contribution, Ethiopia, Healthcare financing, Successes, Universal health coverage

## Abstract

**Background:**

High burden of healthcare expenditure precludes the poor from access to quality healthcare services. In Ethiopia, a significant proportion of the population has faced financial catastrophe associated with the costs of healthcare services. The Ethiopian Government aims to achieve universal health coverage (UHC) by 2030; however, the Ethiopian health system is struggling with low healthcare funding and high out-of-pocket (OOP) expenditure despite the implementation of several reforms in health care financing (HCF). This review aims to map the contributions, successes and challenges of HCF initiatives in Ethiopia.

**Methods:**

We searched literature in three databases: PubMed, Scopus, and Web of science. Search terms were identified in broader three themes: health care financing, UHC and Ethiopia. We synthesised the findings using the health care financing framework: revenue generation, risk pooling and strategic purchasing.

**Results:**

A total of 52 articles were included in the final review. Generating an additional income for health facilities, promoting cost-sharing, risk-sharing/ social solidarity for the non-predicted illness, providing special assistance mechanisms for those who cannot afford to pay, and purchasing healthcare services were the successes of Ethiopia’s health financing. Ethiopia's HCF initiatives have significant contributions to healthcare infrastructures, medical supplies, diagnostic capacity, drugs, financial-risk protection, and healthcare services. However, poor access to equitable quality healthcare services was associated with low healthcare funding and high OOP payments.

**Conclusion:**

Ethiopia's health financing initiatives have various successes and contributions to revenue generation, risk pooling, and purchasing healthcare services towards UHC. Standardisation of benefit packages, ensuring beneficiaries equal access to care and introducing an accreditation system to maintain quality of care help to manage service disparities. A unified health insurance system that providing the same benefit packages for all, is the most efficient way to attain equitable access to health care.

**Supplementary Information:**

The online version contains supplementary material available at 10.1186/s12913-022-08151-7.

## Background

High-performance health financing (HPHF) for universal health coverage (UHC) is adequate and sustainable funding with sufficient risk pooling to spread the financial risk of ill-health to assure the desired levels of service coverage [1]. The strategies designed to increase the share of total health spending for all nations that have made substantial progress towards UHC predominantly rely on compulsory funding sources [2, 3]. Compulsion and subsidisation in health financing are guiding principles for health financing policy to speed up the path toward UHC [4]. Despite increasing the overall share of spending from compulsory sources being important for the progress towards UHC, the ways of pooling arrangements for the prepaid fund matter [5]. A given level of funding organised into fewer pools has more redistributive capacity than the same level of funding organised into more pools [4, 5]. Even though the UHC goals and intermediate objectives are broadly shared, each country's starting point and context for health financing are unique [4]. There was no single best health financing model for UHC for all countries. Thus, the best option for a country in a given circumstance may not be relevant for another country [4].

Universal health coverage provides equitable and quality promotive, preventive, curative, rehabilitative and palliative health services to all people without financial hardship [6–8]. In 2015, the Ethiopian Government was introduced a 20-year plan to achieve UHC [9]. The three pillars of the current health policy of Ethiopia targeted for UHC include the development of an equitable and acceptable standard of health service, assurance of accessibility of health care for all, and provision of health care for the people on a scheme of payment with special assistance mechanisms [10, 11]. Line-item budget, per capita (capitation), and fee-for-services are among Ethiopia's common payment methods for health care services [12]. Community-based health insurance (CBHI) and social health insurance (SHI) are also alternatives to user fees to access equitable healthcare without financial hardship [13]. Both CBHI and SHI provide free-to-access care in public health facilities, reimbursed through a fee-for-service system. However, in Ethiopia, SHI is not implemented concerning the civil servant’s resistance to paying 3% of their salary [14]. Primary healthcare services have also been delivered free of charge or exempted to all service users irrespective of their income level in Ethiopia [15].

Multiple sources finance Ethiopia’s healthcare sector, including loans and donations from all over the world (46.8%), the Ethiopian Government (16.5%), out-of-pocket (OOP) payments (35.8%), and others (0.9%) [16]. The proportion of health financing from domestic sources (excluding the contribution from donors) has increased from 53% of United States dollars (US$) 1.3 billion in 2008 to 78% of US$ 2.7 billion in 2017 [17]. The country’s total health expenditure also rose to nearly Ethiopia Birr (ETB) 50 billion (over US$2.5 billion) in 2013/14 from ETB 1.45 billion (US$230 million) in 1995/96. The per capita health expenditure reached (US$28.65) in 2013/14 from a mere (US$4.5) in 1995/ 96 [18]. But the amount is still meagre compared with the World Health Organization (WHO) recommendation of US$60 per capita spending for delivery of essential health services by 2015 [19]. Ethiopia's health spending constituted 5.6% of the gross domestic product (GDP) in the last decade [18, 20]. For example, the total annual budget of 2019/20 allocated to health was only 5.3% [21], which was less than the average 7% of WHO estimation for low-income countries (LICs) [22].

Despite various HCF reforms in Ethiopia, the country's healthcare system has suffered from low healthcare funding and high OOP payments [17, 23]. However, comprehensive HCF evidence is still lacking that can have implications for achieving UHC and designing strategies to address the gaps in health financing in Ethiopia. Therefore, this review aims to explore the successes, contributions and challenges of HCF toward UHC in Ethiopia.

## Methods

### Ethiopia’s healthcare delivery system

**Fig. 1:**
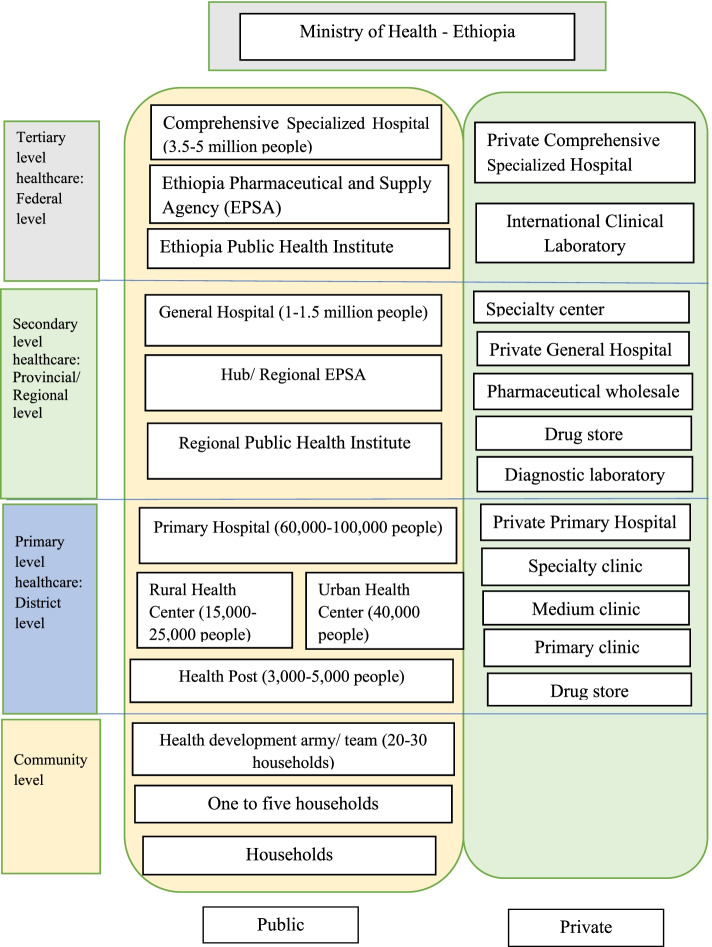
Health care system of Ethiopia

Ethiopia’s health service is structured into a three-tier system: primary, secondary and tertiary levels of care. A primary health care unit (PHCU) comprises four health centers (HCs), five health posts within each health center, and a primary hospital. Each health post is responsible for a population of 3,000–5,000 people. A health center provides both preventive and curative services. In addition to what an HC can provide, a primary hospital provides emergency surgical services, including caesarean section and gives access to blood transfusion services. Secondary level of care consists of general hospitals. In addition, it serves as a referral center for primary hospitals. Finally, the tertiary level of care comprises federally-run, specialised hospitals and university hospitals [11, 16] (Fig. 1).

### Design

A structured narrative review was conducted by reviewing studies published between January 1998 and August 2021 using the three inter-linked HCF framework components.

### Search strategy

Electronic databases such as PubMed, Scopus and Web of Science were used to search all published articles. In addition, hand search engines, such as Google search engine and Google Scholar, were also used to search for additional literature on 08 August 2021. The search terms were identified under three themes: healthcare financing, universal health coverage, and Ethiopia. The search strategies were built based on using the “Medical Subject Headings (MeSH)” and “All field” by linking “AND” and “OR” Boolean operator terms as appropriate (Supplementary file 1). In addition, we used the enhancing transparency in reporting the synthesis of qualitative research (ENTREQ) checklist for reporting the findings [24].

### Inclusion and exclusion criteria

All retrieved studies were initially imported into the Endnote library to remove the duplicates. After removing the duplicates, we screened the articles by title and abstract based on the eligibility criteria. A quality assessment was done for all eligible retrieved articles by the three independent reviewers using the Joanna Briggs Institute’s (JBI) critical appraisal checklist for qualitative research. Later we discussed in the team, and the last author verified the list of studies. We retained the full texts of all relevant studies found to meet the inclusion criteria for the final synthesis (Table 1).

**Table 1:** Eligibility criteria of articles on healthcare financing towards UHC in Ethiopia, 2021

Inclusion criteria	Exclusion criteria
All published articles from January 1998 to August 2021 were included	Articles were excluded from the review, those published before 01 January 1998 and those articles published after the date of submission, preprint?
Articles published in English were included	Articles published in languages other than English were excluded
All articles on healthcare financing towards universal health coverage were included irrespective of the type of articles and methodology	Articles that are not identified the successes or challenges of HCF towards UHC were excluded

### Data extraction and synthesis

We used Microsoft excel spreadsheet format for data extraction. The excel sheet contained the first author's name, year of publication, title, type of article, and its primary outcomes. In addition, we conducted a double check-up and verification of the extracted information. In this review, we assessed the application of HCF to attain UHC, which includes the provision of equitable and quality health services without financial risk. We used framework analysis to synthesise our extracted data using the three inter-linked HCF framework components: revenue generation, risk pooling/ sharing, and strategic purchasing of services [8, 25]. Explanations of the three inter-linked HCF functions were also presented (Table 2).

**Table 2:** Explanations of HCF functions in Ethiopia, 2021

HCF functions	Explanations
Revenue-generation/ mobilisation	Raises the financial resources needed to develop and run a health system. Contributions typically come from individuals/households, firms, and sometimes external sources in the form of development assistance for health
Risk pooling/ sharing	Requires decisions about whether and how financial contributions to the health system are spread across individuals to reduce the financial risk associated with unexpected illness and medical expenses
Purchasing of health services	Requires decisions about how the available funds should be used to purchase (provide) health services (prevention, promotion, treatment, rehabilitation, palliation) and essential public health functions such as population-based promotion and prevention, outbreak readiness and response, and health system governance

## Results

### Description of the reviewed articles

**Fig. 2:**
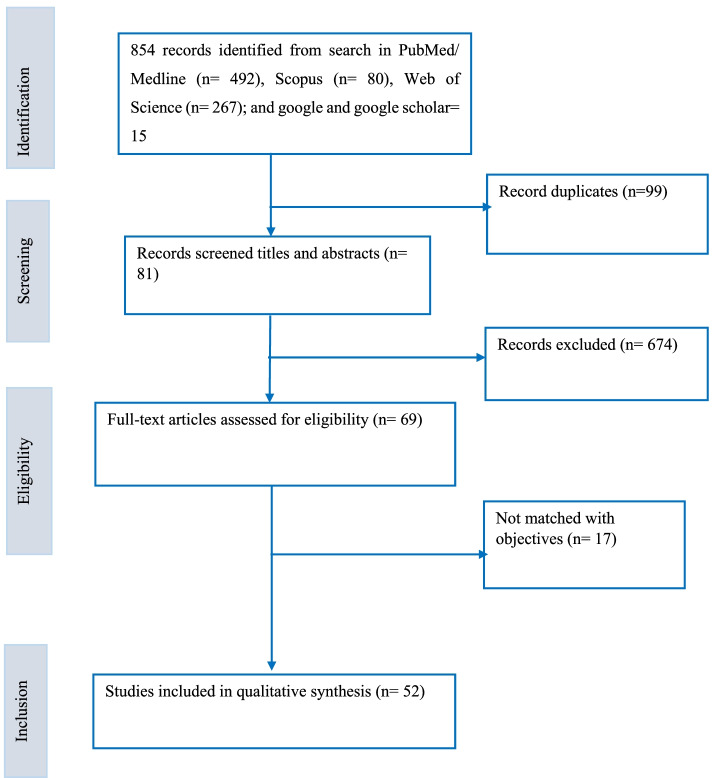
ENTREQ flow diagram to select articles for HCF towards UHC, 2021

Total of 52 articles (32 quantitative, five qualitative, seven mixed, seven project briefs reports, and 1 policy brief) were eligible for final review (Fig. 2). In addition, we included seven articles in Oromia, 13 in Amhara, six in SNNP, seven in Addis Ababa and the remaining in other regions of Ethiopia.

### Successes, contributions and challenges of HCF in Ethiopia

We extracted data from all eligible articles related to the contributions and challenges of HCF towards UHC in Ethiopia. We mapped the key successes, contributions and challenges of the eight HCF reforms/initiatives under the three inter-linked HCF framework components. The successes, contributions and challenges of each HCF function towards UHC are indicated (Table 3).

**Table 3:** Successes, contributions and challenges of healthcare financing towards UHC in Ethiopia, 2021

HCF functions	Reforms	Successes	Contributions	Challenges
Revenue generation	Revenue retention and utilisation	•Retain revenue is an additive to the government health budgets with major sources of revenue including: sale of drugs and other medical supplies, fees for consultation, non-medical services [26]		•Lack of training for governing bodies, slow decision-making, inadequate financial skills by health facility staff, and difficulty interpreting the guidelines [26] •Weak monitoring and evaluation system to conduct regular auditing and make corrective measures [11]
	Private wing in public hospitals	•Offer additional income for health facilities [27]	•Generate additional income for health facilities [28–30]	•Low client satisfaction is associated with their expectations and high payments [31, 32]
	Health insurance	•Mobilise financial resources, increasing revenue generation [33–35]	•Options to generate sustainable resources for health sectors [11, 36]	•Low awareness and costs of premium collection in relation with scattered settlement of agricultural households and mobility of the pastoralists [11, 13, 37] •Low enrolment rates, high dropouts and weak premium collection for the scheme were additional barriers to CBHI resource mobilisation at the community level [11, 38]
	Health facility governance autonomy		•Boards facilitate linkage with the community and advocate increasing resource mobilisation for facilities and solving local problems [39]	•Limited capacities in knowledge and skills for planning, implementing and monitoring health financing [11]
Risk pooling /sharing	User fee settings and revisions	•Promotes cost-sharing between the Government and users considering the community’s willingness, ability to pay and cost of services [27]	•Affordable fees and some sort of subsidy by the Government enhance access to health care [11]	•Variations in regional laws in terms of mandating the user fee revisions and settings. For instance, the mandate of user fee revisions and settings in Amhara and Oromia gave to the regional council by the regional Government. At the same time, SNNP allowed health facilities to introduce user fee revisions [11, 26, 27] •Discrepancy in adherence to regional legislation was another challenge. For example, the regional law gave the mandate of user fee revision to the regional council in the Amhara region, but some health facilities revised user fees on their own [27]
	Health insurance	•Health insurance helps the population with special assistance mechanisms for those who cannot afford to pay [11] •Helps risk pooling and social solidarity for the non-predicted illness [40, 41]	•Contribute to financial risk protection to the users [11, 36] •Contribute to protecting rural dwellers from facing financial hardship to achieve UHC [42] •Contributed to increasing financial risk protection and ensuring UHC for all [43, 44] •Reduce out-of-pocket expenditure (OOP), which increases protection from catastrophic health expenditure [33–35] •Establish financial protection equitably and sustainably for all citizens [29] •Enhance healthcare access and reduce the burden of OOP expenditure as a means of achieving UHC [14, 45]	•Low quality health service; long bureaucracy in reimbursement for institutions and high burden of payroll contributions for SHI [13] •Under coverage of the poor [11] •Unable to pay the premium; inadequate benefit packages; and preference for OOP payment [46] •Voluntary participation in the CBHI scheme results in adverse selection. For instance, households with chronic diseases within their family members purposely enrolled on the CBHI scheme associated with their disease status [47] •Premium load for CBHI is only decided based on family size without considering their income level [48] •High premium contribution, unclear benefit packages, high cost of living and burden of other deductions from salary for SHI [49, 50] •High SHI contribution might lead us to further crisis and illness associated with being unable to wear clean clothes and eat right [49] •Low contract renewal rate related to the inability to afford the premiums and expected returns from the insurance [51, 52] •Free health care services for healthcare providers from their employer health care institution [53]
Strategic purchasing of services	Revenue retention and utilisation	•Increase resource availability for service provision [11] •Use of retained revenue for procurement of drugs and medical supplies, and oversight implementation [27]	•Improve infrastructures, utilities, procure medical equipment, supplies, medical supplies, drugs, information systems, management procedures, and training to enhance services quality [26, 29, 54] •Avail of essential medicines; reduce stock-outs of essential drugs; improve the diagnostic capacity of health facilities; maintain continual quality of care; improve water supply, electricity to health facilities; and health infrastructures [27, 54]	•Lack of understanding of the working procedures and fear of accountability led health facilities to be reluctant to use the retained revenues. This led to health facilities being reluctant to use the retained revenues and demonstrated the loss of efficiency in health service delivery [11]
	Systematising fee-waivers	•Provide free of charge to the poorest segments of the population to access the full range of health services [29]	•Access free health care for poor households [27] •Contribute to increasing financial protection and ensuring UHC for all in Ethiopia [43] •Reduce inequities in access to health care services [29] •Increase healthcare service utilisation for the poor [55]	•Shortage of drugs and procedures in a public health facility; and fee waiver certificate restricted or valid only in a single health facility precludes the use of services for the users [56] •Under-coverage of the poorest; inclusion of those able to pay; and delay or non-reimbursement of costs to health facilities [11] •High non-medical costs, referral to a higher-level facilities, and health care costs including transportation, lodging, food, and opportunity costs [56] •Provision of identification cards during emergency cases may create a loophole for abuse as it is out of schedule [57] •Lack of adequate training on procedures of fee waivers [58] •Lack of consistency and common understanding of selection criteria [57, 58] •Guideline did not verify the income in proportion to the family size [57, 58] •Renewing the waiver card without revising their current economic status resulted in the non-poor receiving benefit intended for the poor [58] •Guideline only considered the income of the family, not their expense for basic needs. For instance, the guideline excluded households with seven members and got ETB 400 per month because of the income. On the other hand, households with four members and earned ETB 300 per month were eligible regardless of the income generated by the family members [57] •Healthcare inequality between fee waivers and cash payers did not protect the poor from financial hardship [58] •Unfair criteria since the criteria could not consider households who had chronic disease family member/s [58] •Absence of a clear income level cut-off for granting fee waivers [59]
	Standardised exemption services		•Provide a package of services free of charge to all citizens through exemptions from fees for certain critical public health services to enhance equity [29] •Provide exempted services include: TB and leprosy diagnosis and treatment; antenatal care; delivery, postnatal care, family planning, leprosy, HIV care, treatment for malaria, immunisation services; HIV/AIDS diagnosis, care and support; and epidemics [11, 60]	•Private health facilities charged for such exempted services to cover the health worker’s time [11] •Shortage of drugs and medical supplies, absence of clear guidance, incurred additional costs, and inadequate support from the Government and NGOs to provide exempted health services [27] •Some health facilities charged for delivery-related services and supplies, such as laboratory services, gloves, glucose, and some drugs, were the challenges in implementing exempted healthcare services [27]
	Private wing in public hospitals	•Offer more choices of services to the users [27]	•Raise motivation of medical professionals; staffs’ sense of hospitals ownership; decline the turnover rate, provide alternative services and improve quality of health services through avail infrastructures and additional investment in staff training [28–30]	•There is no reward for staff based on performance, equity-related complaints on payment, and low knowledge about private wings medical service seekers as alternative options [28] •Poor health care services, access, physical facility, provider behaviors, high expectation and long travel time [61] •Affect work performance of professionals associated with their participation [62]
	Outsourcing of non-clinical services	•Encourages public hospitals to outsource non-clinical services such as laundry, security, and catering by contracting with local vendors that have a comparative advantage in providing these services assisted the hospital in improving its efficiency and reducing the burden on hospital management teams [29]	•Helps to improve efficiency, reduce costs, and enable health facilities to focus on their core clinical services [27] •Controlled cost, reduced the internal administrative burden, increased the effectiveness and quality of the outsourced services [63]	•Conflicts between the hospitals and service providers regarding the quality of non-clinical services, poor specification in the contract, managing the price variations over the life of the contract agreement; and increases in input prices for the cost of the outsourced services were the challenges in implementing outsourcing of non-clinical services [63] •Absence of competitive vendors, limited internal capacity to prepare technically feasible contracts, weak record-keeping and data management systems by hospitals prevented hospitals from documenting the overall achievements, cost–benefit gains and losses from outsourcing [63]
	Health insurance		•Health insurance improves healthcare delivery [11] •Improve access to health care for all citizens [29] •Reduce inequalities in access to basic health care services [11, 36] •Contribute to essential drugs and good perception of quality of care and treatment choice [64, 65] •Increase utilisation of health-care services [40, 41] and improve quality of life [66] •Enhance healthcare access to achieve UHC [14, 45] •Guarantee dwellers of rural areas access to quality health services and achieve UHC [42] •Enhance access to health care and improve health care quality, increasing healthcare utilisation and patient satisfaction [33–35]	•Service disparity between cash payers and insurance users; low-quality health services; inadequate equipment and staff; lack of trained personnel; adverse selection; moral hazard; fraud and corruption [13] •Health facilities are unable to fulfil the criteria to provide healthcare services for insurance beneficiaries [67] •Demands extraordinary drugs; tend to collect more drugs; giving their card to non-insured, and frequent health facility visits were the clients' side moral hazard practices [68] •Overestimating the cost of services to CBHI members, occasional charges of undelivered insurance services, and health providers insulting service users were also the moral hazard of service providers [68] •Exclusion of family members above 18 years did not consider the society’s real situation [49] •Low awareness, low benefits packages, poor perception of quality of services and lack of trust of the management [69–72]
	Health facility governance autonomy	•Existence of clear action plans, national scope of implementation, and regulatory frameworks facilitated HCF [73] •Improve health service quality, introduce accountability and transparency mechanisms [26]	•Ensure facilities' HCF implementation is efficient and effective [29] •Instrumental to improve health facility performance [39] •Allocate resources, bridge performance and improve quality to achieve better health outcomes [74]	•Absenteeism, inappropriate delegation, and lack of adequate priority, capacity, and confusion on the governing body's role [26] •High turnover of governing body/board members [11, 27]

### Revenue generation

Of the reviewed articles, 16 described their findings on revenue generations. Various successes were reported in revenue generation/ resource mobilisation. Health facility retained revenue is an additive to the public health facility budgets with major sources including: sale of drugs, medical supplies, consultation fees, non-medical services [26]. Private wing at hospitals also offers additional income for health facilities [27]. Health insurance is another option to mobilise financial resources, increasing the revenue generation of the health institutions [33–35].

Health financing strategies in Ethiopia also contribute to revenue generation. Private wings at hospitals generate additional income for health facilities [28–30] to provide healthcare services. Health insurance is also another option to generate sustainable resources for health sectors [11, 36]. Health facility governance boards facilitate the linkage of the community to increase resource mobilisation for facilities to solve problems locally [39].

Limited capacity in knowledge and skills of health facility governance related to lack of training and monitoring at federal, regional and woreda levels for planning, difficulty in interpreting the guideline, implementing, and monitoring the HCF [11, 26] were the challenges for revenue generation and utilisation. Low client satisfaction associated with their expectations and high payments were also challenges during private wing implementation [31, 32]. Poor community awareness and costs of premium collection for CBHI concerning the scattered settlement of agricultural households and mobility of the pastoralists were the challenges in revenue collection [11, 13, 37]. Low enrolment rates, high dropouts and weak premium collection for the scheme were additional barriers for CBHI at the community level [11, 38].

### Risk pooling/ sharing

In this review, 24 articles reported their findings on risk pooling/ sharing in healthcare services. Health financing initiatives in Ethiopia have various successes in risk pooling. User-fee-setting and revision promote cost-sharing between the Government and users, considering the community’s willingness, ability to pay and cost of services [27]. Health insurance helps the population with special assistance mechanisms for those who can not afford to pay [11]. Health insurance can also help risk pooling/ sharing and social solidarity for the non-predicted illness [40, 41].

Health financing strategies in Ethiopia also contributes to risk pooling. Fee-setting and revision contribute to setting affordable fees and some subsidy by the Government to enhance healthcare access [11]. Health insurance can contribute to financial risk protection for the users [11, 36]. Community-based health-insurance particularly contributes to protecting rural dwellers from facing financial hardship to ensure UHC for all [42–44]. It reduces out-of-pocket expenditure (OOP) for healthcare services, reducing catastrophic health expenditure [33–35]. Health insurance can also establish financial protection equitably and sustainably for all citizens to enhance healthcare access and achieve UHC by reducing OOP expenditure for healthcare services [14, 29, 45].

Variations in regional laws and discrepancies in adherence to regional legislation in terms of mandating the user fee revisions and settings were the challenges to maintaining the consistency of HCF in Ethiopia. For instance, the mandate of user fee revisions and settings in Amhara and Oromia gave to the regional council, while SNNP allowed health facilities to introduce user fee revisions [11, 26, 27]. Moreover, the provincial law gave the mandate of user fee revision to the provincial (regional) council in the Amhara region, but some health facilities revised user fees on their own [27]. The adverse selection associated with voluntary based CBHI, inability to afford the premium, inadequate benefit packages; preference for OOP payment, and considering only family size without their level of income for premium load [46–48] were the challenges during CBHI implementation. The constraints of implementing SHI are the high burden of payroll contributions to SHI, long bureaucracy in reimbursement, under-coverage of the poor, low contract renewal rate and expected returns [13, 51, 52]. Unclear benefit packages, high cost of living and the burden of other deductions from salary were the obstacles to initiating SHI [49, 50]. Free health care services for healthcare providers and frustration of illness associated with high SHI contribution [49, 53] could be the challenges to introducing SHI.

### Purchasing of healthcare services

Forty articles from the reviewed articles described the strategic purchasing of healthcare services. Health financing initiatives in Ethiopia have various successes in purchasing healthcare services. Revenue retention can increase resource availability for service provision by using retained revenue to procure drugs and medical supplies and implement oversight [11, 27]. Fee waiver system provides free of charge to the poorest segments of the population to access the full range of health services [29]. Public hospitals' private wings offer more services to the service users [27]. Outsourcing non-clinical services at public hospitals encourages hospitals to outsource non-clinical services, such as laundry, security, and catering, by contracting with local vendors that improve the hospital’s efficiency and reduce the hospital management burden [29]. A clear action plan for healthcare delivery and the introduction of accountable and transparent regulatory frameworks facilitate HCF implementation [26, 73].

Health facility revenue retention could improve infrastructures, diagnostic capacity, procure medical equipment, supplies, medical supplies, drugs, information systems, management procedures, water supply, electricity, and training to enhance quality of healthcare services [11, 27, 54]. It assisted in availing essential medicines; reduce stock-outs of essential drugs; improve health facilities' diagnostic capacity, maintaining continual quality of care; improving water supply, electricity to health facilities; and health infrastructures [27, 54]. Exempted healthcare also helped to deliver the package of services free of charge to all citizens for certain critical public health services to enhance coverage and equity [29]. These services include TB and leprosy diagnosis and treatment; antenatal care; delivery, postnatal care, family planning, leprosy, HIV care, treatment for malaria, immunisation services; HIV/ AIDS diagnosis, care and support, and epidemics [11, 60]. In addition, outsourcing non-clinical services at public hospitals assisted to improve hospitals’ efficiency, reduce internal administrative burden, control costs, enable health facilities to focus on core clinical services, and increase effectiveness and quality of services [27, 29, 63].

Access to healthcare services free health care for poor households through fee waiver systems increases financial protection and ensures UHC for all [27, 43]. Fee waiver systems increase healthcare service utilisation and reduce inequalities in accessing healthcare services for the poor [29, 55]. Private wing at hospitals can raise the motivation of medical professionals, staffs’ sense of hospitals ownership and a decline the turnover rate could help offer more choices and quality of care [27–30]. Health insurance is reduced inequitable healthcare services for all citizens and improves healthcare delivery [11, 29, 36] and facilitates the path toward UHC [14, 45]. Community-based health insurance also contributes to avail essential drugs, good quality of care and treatment of choice to the users [64, 65]. Health facility governance autonomy is instrumental in increasing facility performance, fair resource allocation and quality of healthcare services to achieve better health outcomes [39, 74]. Community-based health insurance could enhance access to healthcare services and quality of life [40, 41, 66]. It also guaranteed access to equitable quality healthcare services among dwellers of the informal and rural areas [33–35, 42].

High non-medical costs, including transportation, lodging, food, and opportunity costs, were the barriers for the poor to access healthcare [56]. Lack of consistency and common understanding of selection criteria, poor training on procedures, low coverage of the poor, inclusion of those able to pay, delay or non-reimbursement of the costs to health facilities, and corruption [11, 57, 58] were also the challenges during fee waiver implementation. Non-consideration of the household's income in proportion to the family size and their expenses for basic needs in the fee waiver guideline were the challenge to select the eligible households [57, 58]. For instance, the guideline excluded households with seven members and got ETB 400 per month. On the other hand, households with four members who earned ETB 300 per month were eligible regardless of the income generated by the family members [57]. The absence of a clear income level cut-off for granting fee waivers; renewal of the waiver card without revision of economic status; healthcare disparity between fee waivers and cash payers; and non-consideration of households with chronic disease within the family were the other barriers to protect the poor from financial hardship [58, 59]. In addition, restriction of fee waiver certification only in a single health facility precludes services for the poor [56]. Shortage of drugs and medical supplies, absence of clear guidance, incurred additional costs, inadequate support, charging for health delivery-related services and supplies by health facilities were also challenges in exempted health services [27].

Lack of understanding of the working procedures and fear of accountability led to health facilities being reluctant to use the retained revenues and demonstrated a loss of efficiency in the healthcare service delivery [11]. The absence of rewarding systems for staff based on performance, poor physical and healthcare infrastructures, provider behaviours, high expectations, and low awareness about alternative options of the private wing at the hospital were the challenges to its utilisation [28, 61, 62]. During its implementation, conflicts between hospitals and service providers regarding the quality of non-clinical services and poor contract specifications were common challenges [63]. Service disparity, low-quality health services, inadequate equipment and staff, lack of trained personnel, adverse selection, moral hazard, fraud, and corruption were the barriers affecting health insurance delivery [13]. Exclusion of family members above 18 years from insurance beneficiaries [49] and health facilities unable to fulfil the criteria to provide the insurance service [67] also challenged healthcare delivery. Demands extraordinary drugs; tend to collect more drugs, and give their card to non-insured patients from the client side while overestimating the cost of services. The charge of undelivered insurance services from service providers' perspective were frequently occurred moral hazards to deliver healthcare services by CBHI [68]. Low awareness and benefit packages, poor perception of quality of health services, and lack of trust of the management [69–72] were the challenges to enrol CBHI membership. High turnover of board members, absenteeism, inappropriate delegation, lack of capacity, and confusion about the governing body's role hampered leadership and community ownership [11, 26, 27].

## Discussion

We explored the successes, contributions, and challenges towards UHC concerning revenue generation, risk pooling/ sharing, and strategic purchasing. Successes of healthcare financing initiatives in Ethiopia include generating an additional income for health facilities, promoting cost sharing between Government and users, risk-sharing for the non-predicted illness, providing special assistance mechanisms for those who can not afford to pay, and purchasing healthcare services [11, 26, 27]. Ethiopia's healthcare financing initiatives have made significant contributions to healthcare infrastructures, medical supplies, diagnostic capacity, drugs, financial risk protection, and healthcare services [27, 29, 55]. On the contrary, poor access to equitable quality healthcare services was the major challenges associated with low healthcare funding and high OOP payments in Ethiopia [17, 23, 75, 76]. Adverse selection, moral hazard, low enrolment in CBHI, poor awareness, lack of consistency and fairness in the implementation of HCF initiatives [11, 13, 38, 48] were also identified as the challenges to access healthcare services and disparity. Furthermore, high non-medical costs, including transportation, lodging, food, and opportunity costs, were barriers to fee waivers and exempted service users from receiving health services [27, 56].

In Ethiopia, health insurance is one of the options/ mechanisms for risk pooling and social solidarity to access healthcare for non-predicted illnesses [40, 41]. Nevertheless, low healthcare funding and high OOP payments [17, 23] were Ethiopia's barriers to health financing. In South Africa, the low risk-pooling and social solidarity could be alleviated by reducing fragments of funding sources and directing all possible sources to a centralised well-managed pool [77]. Centralisation in funding sources is essential and critical to providing equity and improving HCF where a single purchaser of health services [78]. Cross-subsidisation is achieved through integrated funding pools because managing separate funds for different groups limited the benefits of cross-subsidies, and it became difficult to merge fragmented pools [77]. A pre-payment scheme, such as SHI funds for formal sectors and direct taxation with the incremental progressive source of HCF, was essential to reduce the burden of OOP payments [79]. Centralisation in funding sources could make Thailand exemplary progress toward UHC with Government subsidisation for the poor [80]. A pro-poor shift of subsidisation is essential where a more progressive HCF mechanism enables equitable health financing and is vital to develop an HCF mechanism to realise UHC [81].

Multiple sources finance Ethiopia’s healthcare sector, including loans and donations, OOP payments (households), and others [16, 82]. But the healthcare expenditure of Ethiopia is among the least compared with the estimated WHO average of LICs (7%) [22] and the Abuja agreement (15%) [83]. This low health care revenue in the country could be associated with low government budgets, corruption, low enrolment and a high dropout rate from CBHI membership [11, 13, 21, 38]. In Malawi, the Debt relief strategy substantially contributes to the country’s steady progress towards the Abuja target [84], making Malawi’s share of GDP to the health sector. It makes Malawi’s share of GDP in the health sector higher than other LICs [85]. In Ghana, refining the premium of the national health insurance (NHI), gradual increment of funds to the health sector, and multi-sectoral advocacy to increase revenues by the Government had a significant impact on the country’s health revenue generation [86]. Thailand has also set a successful, equitable and feasible HCF tax procedure for most of its population [87]. The general tax method was the most progressive source of finance for healthcare. It utilises the finance ministry's existing resources, expertise, and mechanisms to enforce and collect the tax payments in Thailand [79]. The tax financing mechanism was a popular and proven healthcare financing and instrumental in achieving UHC in countries with low tax. Progressive tax practices and a pro-poor tax framework for capital gains and profits might support achieving global health objectives [88].

Healthcare service disparity, adverse selection, low-quality health services, moral hazard, fraud and corruption were the challenges in CBHI implementation in Ethiopia [13]. A study conducted in Southeast Asia and the People’s Republic of China showed that service inequality could be narrowed by applying a -payer health insurance system with a unified benefit package to provide more equitable healthcare services [89]. Client-side moral hazard could also be reduced through a cost-sharing mechanism, such as the co-payment approach, which diminishes non-urgent healthcare visits [90, 91]. Mandatory CBHI was pivotal in setting Rwanda toward UHC and making the country with the highest enrolment in health insurance in sub-Saharan Africa (SSA) [92]. A study conducted in Ethiopia also recommended that a mandatory CBHI approach was essential to address the challenges of adverse selection associated with voluntary participation [47]. Standardisation of benefit packages, ensuring equal access to care, and introducing an accreditation system to ensure the quality of care was also helpful in managing service disparities [93]. High non-medical costs, including transportation, lodging, food, and opportunity costs, were the deterrents for fee waivers and exempted service users to access healthcare services [27, 56]. This high non-medical cost precludes the poor from healthcare service utilisation. In LICs, studies recommended that the poor be waived for user fees and reimbursed, particularly for fee waivers for their access costs to health care, including transportation, lodging, food, and opportunity costs [94, 95] solve the accompanying financial catastrophic shock.

### Policy implications

The policy implications for this review focused on the contribution and challenges of the HCF reforms for future improvement in the country. Appropriate health financing strategies that safeguard financial risk protection underpin sustainable health services and attain UHC. This review also has an implication to provide more equitable clinical healthcare services or ensure equal access to healthcare in general. This finding will also help to ensure the quality of care and address the clinical healthcare service disparities in Ethiopia. It advances awareness among health programmers and policymakers about the importance of HCF as a key building block of the health system. It also provides an insight on the critical HCF reforms in Ethiopia, include: revenue retention and utilisation, a fee-waiver system for the poor, exemption services, user fee setting and revision, private wing in public hospitals, outsourcing non-clinical services, health insurance, and promoting health facility governance autonomy to improve its implementation on the health system. The review could help the policymakers and government officials to revise and update its HCF strategies.

### Strengths and limitations

This review provides ample evidence on HCF towards UHC in Ethiopia. On the contrary, this review did not include a meta-analysis to estimate the pooled effect of HCF on the progress of healthcare service delivery towards UHC in Ethiopia since a meta-analysis to be valid requires all included studies to be sufficiently similar. However, a narrative review can cover a wide range of subject matter at various levels of completeness and comprehensiveness that may consist of research findings.

## Conclusion

The healthcare financing initiatives in Ethiopia have had various successes, including generating additional income for health facilities and promoting cost-sharing between Government and users. Risk-sharing/ social solidarity for the non-predicted illness, providing special assistance mechanisms for those who can not afford to pay, and purchasing healthcare services were also the successes of Ethiopia’s health financing. Ethiopia's health financing has significant contributions to healthcare infrastructures, medical supplies, diagnostic capacity, drugs, financial risk protection, and healthcare services. However, poor access to equitable and quality healthcare services was associated with low healthcare funding and high OOP payments in Ethiopia. Health service disparity, adverse selection, moral hazard, low enrollment in CBHI, poor awareness, fraud and corruption were also barriers to health service delivery. The restriction of fee waiver certificates to a single health facility shall be revised to keep the country's referral loop to facilitate UHC progress. Mobilising domestic and external resources, aligning donor funding into the government system, and evidence-based allocation of available resources are essential to advance HCF systems. Standardising benefit packages, ensuring equal access to care, and introducing an accreditation system to maintain quality of care are also helpful in managing service disparities. Therefore, a robust health care financing system is required to speed up the path towards UHC.

## Supplementary Information

**Additional file 1. **PubMed search strategy.

## Data Availability

Not applicable.

## Abbreviations

ENTREQ: Enhancing Transparency in Reporting the Synthesis of Qualitative research
HCF: Health Care Financing
JBI: Joana Brig's Institute
NGOs: Non-Governmental Organisations
UHC: Universal Health Coverage
SDGs: Sustainable Development Goals
WHO: World Health Organization
