# Supplementary material for: Contributions and challenges of healthcare financing towards universal health coverage in Ethiopia: a narrative evidence synthesis
Source: BMC Health Serv Res. 2022 Jul 5;22:866. doi: 10.1186/s12913-022-08151-7 (PMC9254595; doi:10.1186/s12913-022-08151-7)
Supplement: Supplementary file 1 — Additional file 1. PubMed search strategy. [file 12913_2022_8151_MOESM1_ESM.docx]

**Search strategy**

1. ((Healthcare financing OR community-based health insurance OR social health insurance OR risk pooling OR risk-sharing OR health insurance OR revenue collection OR resource mobilisation OR revenue retention OR resource utilisation OR purchasing of services OR fee waiver OR fee setting OR fee revision OR exempted services OR outsourcing of non clinical health services OR private wing at hospitals OR autonomy of health facilit*) AND (universal health coverage OR universal access OR equit* OR qualit* OR risk protection OR financial hardship OR responsive* OR efficien* OR effective*) AND (Ethiopia))
